# Identification of danthron as an isoform-specific inhibitor of HEME OXYGENASE-1/cytochrome P450 reductase interaction with anti-tumor activity

**DOI:** 10.1186/s12929-018-0411-y

**Published:** 2018-01-23

**Authors:** Yi-Tai Chou, Fu-Fei Hsu, Dun-Yao Hu, Ying-Chih Chen, Yuan-Hao Hsu, John T.-A. Hsu, Lee-Young Chau

**Affiliations:** 1 Institute of Biomedical Sciences, Academia Sinica, No.128, Sec. II, Academy Rd. Taipei 115, Taipei, Taiwan; 2 Department of Chemistry, Tunghai University, Taichung, Taiwan; 3 Life Science Research Center, Tunghai University, Taichung, Taiwan; 4 Institute of Biotechnology and Pharmaceutical Research, National Health Research Institutes, Zhunan, Miaoli County, Taiwan

**Keywords:** Heme oxygenase-1, Cytochrome P450 reductase, Protein-protein interaction, Anti-cancer drug

## Abstract

**Background:**

Heme oxygenase (HO) catalyzes NADPH-dependent degradation of heme to liberate iron, carbon monoxide and biliverdin. The interaction between HO and cytochrome P450 reductase (CPR), an electron donor, is essential for HO activity. HO-1 is a stress-inducible isoform whereas HO-2 is constitutively expressed. HO-1 induction is commonly seen in cancers and impacts disease progression, supporting the possibility of targeting HO-1 for cancer therapy.

**Methods:**

We employed a cell-based bioluminescence resonance energy transfer assay to screen compounds with ability to inhibit HO-1/CPR interaction. The effect of the identified compound on HO-1/CPR interaction was confirmed by pull down assay. Moreover, the anti-tumorigenic activity of the identified compound on HO-1-enhanced tumor growth and migration was assessed by trypan blue exclusion method and wound healing assay.

**Results:**

Danthron was identified as an effective small molecule able to interfere with the interaction between HO-1 and CPR but not HO-2 and CPR. Additional experiments with structural analogues of danthron revealed that the positions of hydroxyl moieties significantly affected the potency of inhibition on HO-1/CPR interaction. Pull-down assay confirmed that danthron inhibited the interaction of CPR with HO-1 but not HO-2. Danthron suppressed growth and migration of HeLa cells with stable HO-1 overexpression but not mock cells. In contrast, anthrarufin, a structural analog with no ability to interfere HO-1/CPR interaction, exhibited no significant effect on HO-1-overexpressing HeLa cells.

**Conclusions:**

These findings demonstrate that danthron is an isoform-specific inhibitor for HO-1/CPR interaction and may serve as a lead compound for novel anticancer drug.

## Background

Heme oxygenease (HO) is a rate-limiting enzyme catalyzing NADPH-dependent oxidative degradation of heme to liberate ferrous ion, carbon monoxide (CO), and biliverdin [1].

The electrons required for HO reaction are provided by cytochrome P450 reductase (CPR), which is colocalized with HO on endoplasmic reticulum [2]. HO plays a vital role in systemic iron homeostasis [1]. The free iron released from erythrocyte-hemoglobin turnover is recycled back to bone marrow for erythropoiesis. Moreover, CO gas is a signaling molecule with diverse biological effects, including anti-inflammation, immune-modulation, proangiogenesis and anti-apoptosis [3]. Biliverdin and its subsequent metabolite, bilirubin, are antioxidants contributing to the cytoprotective function of HO [3, 4].

Two HO isoforms were identified in mammalian system [1]. In contrast to HO-2 which is constitutively expressed in various tissues and cells, HO-1 is a stress-responsive isoform highly expressed in many disease states, including cancer [4]. Over the past decade, considerable evidence has revealed the pathological role of HO-1 in cancer progression [4]. HO-1 overexpression facilitates cancer cell growth and survival post irradiation or genotoxin treatment. It also promotes angiogenesis and metastasis through modulating cancer microenvironment. Studies have demonstrated that depletion of HO-1 expression by specific gene knockdown approach or inhibition of HO-1 activity by competitive inhibitors, such as zinc protoporphyrin IX, increases tumor sensitivity to chemotherapeutic agent or irradiation-induced cell death and suppresses cancer metastasis [5–8]. It is conceivable that small molecules with specificity to block HO-1 activity would offer new therapeutics for treating this devastating disease [9–11].

Recently, increasing evidence has supported that the small molecule inhibitors of protein-protein interaction are promising therapeutic targets [12–14]. Early studies have shown that HO-1 interacts with CPR and mutations of the amino acid residues in the interface leads to reduced HO activity [15–17], suggesting that small molecules interfering HO-1/CPR interaction may act as a potent inhibitor of HO-1 reaction. To explore this possibility, in the present study we employed a cell-based bioluminescence resonance energy transfer (BRET) assay [18, 19] to screen a compound library and identify molecules interfering the interaction between HO-1 and CPR. This approach allowed us to identify a few compounds with inhibitory effect on HO-1/CPR interaction to various degrees. Among the initially identified ten compounds, danthron (1,8 dihydroxyanthraquinone) exhibited dose-dependent effect without cytotoxicity at the highest concentration tested. Moreover, danthron is a structurally simple compound and its structural analogues are available for analysis. Therefore, in the present study we further characterized the potencies of danthron and structural analogues on HO-1/CPR interaction. Interestingly, the interaction between HO-2 and CPR was not significantly affected by danthron and structural analogues. We also performed experiments to assess the inhibitory effect of danthron on HO-1-induced cancer cell growth and migration.

## Methods

### Reagents

The library of natural product collection was obtained from MicroSource Discovery systems Inc. The 96-well plates for bioluminescent signal detection were purchased from PerkinElmer Life Sciences. Coelentarazine 400a was from Gold Biotechnology. Anti-His tag, anti-β-actin and anti-green fluorescent protein (GFP) antibodies were from Gene Tex. Anti-CPR and anti- glyceraldehyde 3-phosphate dehydrogenase (GAPDH) antibodies were from Abcam. Anti-GFP-affinity Sepharose was obtained from Abcam. Danthron (1,8 dihydroxyanthraquinone), 1,4,5-trihydroxyanthra-9,10-quinone, 1-hydroxyanthra-9,10-quinone, and anthrarufin (1,5-dihydroxyanthraquinone) were purchased from Sigma-Aldrich.

### Plasmid constructs

The N-terminal Flag-tagged-HO-1 plasmid was prepared as described previously [20]. To prepare HO-1(Luc-HO-1) and HO-2 (Luc-HO-2) constructs with their N-termini fused to a luciferase construct, human HO-1 and HO-2 cDNAs were subcloned into the pcDNA-RLuc8 vector. To construct a human CPR cDNA with C-terminus fused to a GFP2 construct (CPR-GFP), CPR and GFP2 cDNAs were subcloned into a pCMV-2 vector sequentially. Both pcDNA-RLuc8 and GFP2 vectors were kindly provided by Dr. Klim King (Genomic Research Center, Academia Sinica, Taipei, Taiwan) [21]. A truncated human HO-1 construct (aa 13–260) and human HO-2 construct (aa 33–288) fused with 6 x His-tag at C-terminus were subcloned into pQE-60 vector as described previously [22].

### Cell lines

HEK293T cells were cultured in Dulbecco’s modified Eagle’s medium (DMEM) supplemented with 10% fetal bovine serum (FBS) and 1% penicillin/streptomycin. HeLa cell lines without (mock) or with stable expression of Flag-tagged HO-1 (HeLa-HO) were established and cultured in DMEM containing 10% FBS and 400 μg/ml hygromycin B as described previously [23].

### BRET assay

For BRET donor saturation assay, HEK293T cells were seeded in 6-well plate (2 × 10^5^/well) and transfected with a constant amount of Luc-HO-1 or Luc-HO-2 plasmid (0.25 μg of DNA) and increasing amounts of CPR-GFP plasmid (from 0 to 0.875 μg of DNA) using GenJet Plus transfection reagent (SignaGen laboratories, MD, USA) according to the manufacturer’s instruction. For BRET inhibition assay, cells were co-transfected with equal amounts of RLuc-HO-1 and CPR-GFP vectors (1:1). At 24 h post transfection, cells were harvested and reseeded into 96 well plates at a density of 2 × 10^4^ cells /well in triplicates. After 18 h, medium was replaced with DMEM containing 0.5% FBS and cells were treated without or with indicated concentrations of tested compounds in culture for 6 h. Plate was then washed twice with phenol-red free MEM containing 5 mM HEPES, followed by addition of 100 μl of the same medium containing 5 μM of coelenterazine 400a into each well. The plate was immediately loaded in SpectroMax Paradigm Detection Platform equipped with a Dual-color luminescence detection cartridge and SoftMax Pro 6.2.2 (Molecular Devices, Sunnyvale CA, USA). The BRET signal was obtained by the sequential integration of the luminescence and green fluorescence detected at 370–410 nm and 500–530 nm, respectively, over 60 to 150 s and calculated as the ratio of light emitted at 500–530 nm to that emitted at 370–410 nm. Graphpad prism software was used to fit the nonlinear curve. The one-site saturation binding model was chosen to create the titration curves for the determination of Bmax (maximum number of binding sites) and K_D_ (ligand concentration that binds to half the receptor sites at equilibrium). The Bmax and K_D_ values were referred to as BRETmax and BRET_50,_ respectively [24, 25]. The IC_50_ value was also determined from the inhibition curve for each compound.

### His-tagged HO-1 protein purification

His-HO-1 and His-HO-2 proteins were induced in *Escherichia coli* (strain JM109) transformed with corresponding pQE-60 vector bearing HO-1 or HO-2 construct and purified by TALON™ metal affinity resin as described previously [22]. The protein purity was examined by SDS-polyacrylamide electrophoresis (SDS-PAGE) and coomassie blue staining.

### Western blot analysis

Cells were lysed in buffer containing 20 mM Tris-HCl pH 7,4, 100 mM NaCl, 1% Triton X-100 and protease inhibitor cocktail, followed by centrifugation at 12,000 xg for 15 min at 4 °C.

Supernatant was removed and protein concentration determined by Bio-Rad protein assay. Cell lysates (20 μg) were subjected to SDS-PAGE followed by immunoblotting with indicated antibodies as described previously [23].

### Pull-down assay

HEK293T cells were transfected with CPR-GFP construct for 24 h. Cells were lysed with buffer A (20 mM Tris-HCl pH 7,4, 100 mM NaCl) containing 1% Triton X-100 and protease inhibitor cocktail. After centrifugation at 12,000 xg for 15 min at 4 °C, supernatant was removed and CPR GFP protein was immunoprecipitated with ant-GFP-affinity Sepharose at 4 °C overnight.

After two washes with buffer A containing 0.1% Triton x-100, the CPR-GFP-bound Sepharose was resuspended in 30 μl of buffer A. Ten μl of CPR-GFP-bound Sepharose was then incubated with 1 μg of His-HO-1 or His-HO-2 in the absence or presence of indicated compounds in 100 μl buffer A at room temperature for 1 h with rotation. The Sepharose was washed for three times with buffer A and bound proteins eluted by 2X SDS sample buffer and then subjected to SDS-PAGE and Western blot analysis using anti-His (1:2000) and anti-GFP (1:2000) antibodies.

### Cell proliferation

Mock and HO-1-overexpressing HeLa cell lines were seeded in triplicate in 12-well plates at a density of 5 × 10^4^ cells/well and grown in complete medium without or with indicated concentrations of tested compounds. At indicated times, cells were harvested and counted by trypan blue exclusion method.

### Wound healing assay

Cell migration was assessed by wound healing assay using the Culture-Inserts (ibidi, Germany). Briefly, HeLa cells stably expressing control vector (mock) or Flag-tagged full-lenth-HO-1 were seeded on both sides of a culture-insert with a 500 μm gap between each side of the well (3.5 × 10^4^ cells/well), and grown for 24 h to reach confluency. Following serum deprivation for 24 h, the inserts were gently removed, and cells were incubated in DMEM complete medium without or with indicated concentrations of compounds. Cells were photographed at insert removal (0 h) and following 24 h of incubation.

### Statistical analysis

Data were expressed as mean ± SE. Student’s t-test was used to compare data from two groups. Analysis of data from more than two groups were conducted by one-way ANOVA followed by multiple comparisons among means (Tukey’s test) using SigmaStat 3.5 (Systat Software, Inc., San Jose, CA, USA). *P* < 0.05 was considered statistically significant.

## Results

### Monitoring HO-CPR interaction by BRET assay

**Fig. 1:**
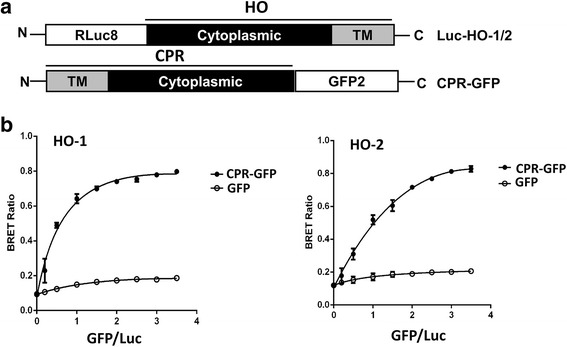
BRET assay monitoring interaction of HO-1 or HO-2 with CPR. **a** The constructs of Luc-fused HO and CPR-fused GFP proteins. **b** HEK 293 T cells were co-transfected with the constant amount of Luc-HO-1 or Luc-HO-2 construct and increasing amounts of GFP or CPR-GFP construct as indicated. After 24 h, cells were reseeded in triplicate in 96-well plates for 18 h. Luciferase substrate was then added into each well and the BRET signal was monitored and expressed as the ratio of signals detected at 515 nm/410 nm. Data shown are mean ± SE of three independent experiments

To first evaluate whether the HO-1 isoform and CPR interaction can be monitored by BRET assay, HEK293T cells were transfected with a fixed amount of vector bearing N-terminal-luciferase-fused HO-1 construct (Luc-HO-1) together with increasing amounts of GFP vector or vector bearing CPR construct with its carboxyl-terminus fused to GFP (CPR-GFP) (Fig. 1a). At 24 h post transfection, BRET analysis was performed. As shown in Fig. 1b, the BRET signals from the cells transfected with GFP and Luc-HO-1 were much weaker and did not significantly affected by the amounts of GFP acceptor. In contrast, the signals obtained from cells transfected with CPR-GFP and Luc-HO-1 vectors were increased and reached a plateau along with increasing amounts of CPR-GFP acceptor, indicating that the BRET signals resulted from the specific interaction between Luc-HO-1 and CPR-GFP. Likewise, the BRET signal was also detected in cells transfected with Luc-HO-2 and CPR-GFP (Fig. 1b), supporting the interaction between HO-2 isozyme and CPR.

### Identification of small compounds interfering interaction of CPR with HO-1 but not HO-2

**Fig. 2:**
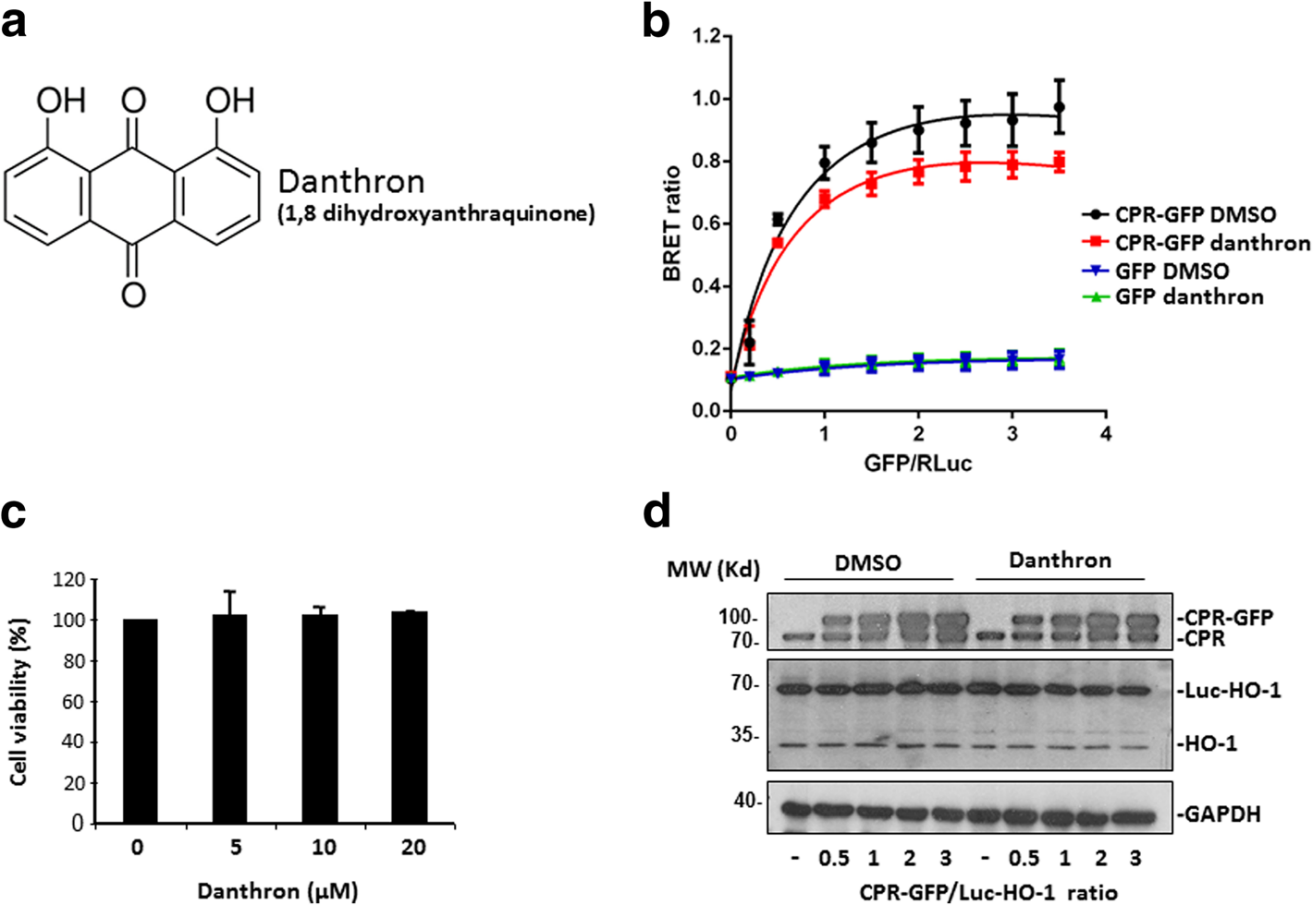
Danthron reduces HO-1/CPR BRET signal without affecting cell viability. **a** Structure of danthron. **b** HEK293T cells were co-transfected with the constant amount of Luc-HO-1 construct and increasing amounts of GFP or CPR-GFP construct as indicated. After 24 h, cells were reseeded in 96-well plates in triplicate for 18 h, followed by treatment with 20 μM danthron for 6 h. Luciferase substrate was then added into each well and the BRET signal was monitored. Data shown are the mean ± SE of three independent experiments. **c** HEK293T cells were treated with indicated concentration of danthron in culture for 6 h. Cell viability was then assessed by trypan blue exclusion method. Data shown are the mean ± SE of three independent experiments. **d** HEK293T cells were cotransfected with increasing ratio of CPR-GFP/Luc-HO-1 constructs for 24 h as described above, followed by treatment without (DMSO) or with 20 μM danthron for 6 h. Cell lysates were prepared and Western blot analysis was performed using antibodies against HO-1, CPR and GAPDH as indicated

**Fig. 3:**
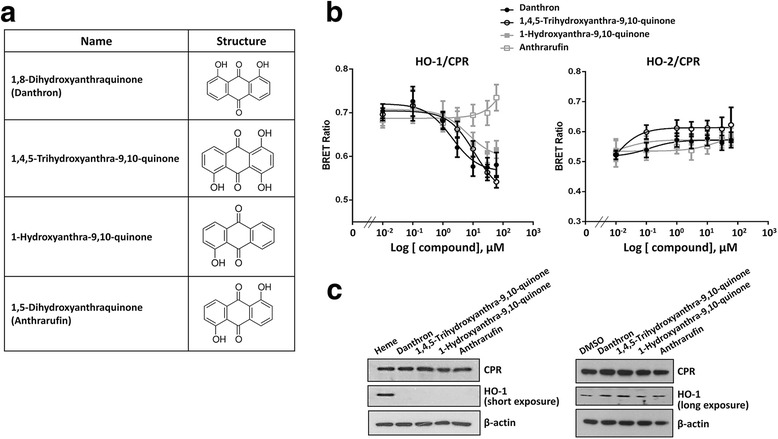
Effects of danthron structural analogs on interaction of HO isozyme with CPR. **a** The structures of danthron analogs. **b** Non-linear regression curves showing the effects of dantron and structural analogs on HO-1/CPR or HO-2/CPR interaction. Data show the mean ± SE of four independent experiments. **c** HEK293T cells were treated with 20 μM of indicated compounds for 16 h. Cell lysates were then prepared and the expression levels of endogenous CPR and HO-1 were examined by Western blot analysis

We then employed the BRET assay in HEK 293 T cells transfected with donor/acceptor vectors at 1:1 ratio, which elicits signal within linear detection, to identify small compounds exhibiting activities to modulate HO-1/CPR interaction in live cells. At 24 h post transfection, cells were reseeded in 96 well plates and treated with tested compounds at a concentration of 10 μM for 6 h prior to BRET assay. From screening the Natural Product Collection (MicroSource Discovery systems Inc.) which contains ~ 700 compounds, danthron (1,8 dihydroxyanthraquinone) (Fig. 2a) was found to significantly decrease HO-1/CPR BRET signal. As shown in Fig. 2b, when the inhibition assay was performed with increasing ratios of acceptor/donor, danthron did not affect the background BRET signal derived from coexpression of GFP and Luc-HO-1 in cells. However, danthron treatment significantly reduced BRETmax from cells coexpressing CPR-GFP and Luc-HO-1 (0.967 ± 0.060 vs 0.771 ± 0.004; *P* < 0.05) but did not significantly affected the value of BRET_50_ (0.582 ± 0.033 vs 0.534 ± 0.053; *P* = 0.719). The effect of danthron was not due to the cytotoxicity (Fig. 2c). Furthermore, Western blot analysis revealed that danthron treatment did not significantly affect the expression levels of CPR-GFP and Luc-HO-1 in cells co-transfected with increasing ratio of CPR-GFP/ Luc-HO-1 vectors or the expressions of endogenous CPR and HO-1 (Fig. 2d). These observations support the effect of danthron on CPR/HO-1 interaction. Further experiment demonstrated that the effect of danthron was dose-dependent with an IC_50_ of 2.087 μM (Fig. 3b). To examine the structural specificity of danthron on HO-1/CPR interaction, we performed the BRET assays with several structural analogs with hydroxyl groups attached to different positions of the phenolic rings (Fig. 3a). As shown in the same figure, the potencies of these analogues were danthron > 1-hydroxyanthra-9,10-quinone (IC_50_: 10.06 μM) ≥ 1,4,5-trihydroxyanthra-9,10-quinone (IC_50_: 10.36 μM) > anthrarufin. Of note, anthrarufin (1,5-dihydroxyanthraquinone) showed no significant effect on HO-1/CPR BRET signal up to 30 μM, but increased BRET signal at higher concentration. To further elucidate whether the effect is isoform specific, we examined the effects of these compounds on interaction of HO-2/CPR. Interestingly, danthron and its analogues at concentrations exhibiting inhibitory effects on HO-1/CPR interaction did not showed significant effect on HO-2/CPR BRET (Fig. 3b). This finding demonstrates that the effects of danthron and analogs are HO isoform specific. To examine whether the inhibitory effects of danthron and its analogues on HO-1/CPR were resulted from the effects on endogenous CPR and HO-1 expression, we performed Western blot analysis of cell lysates harvested from HEK293T cells treated with 20 μM of heme, an HO-1 inducer, and indicated compounds for 16 h in culture. As demonstrated in the left panel of Fig. 3c, HO-1 was highly induced by heme but not by danthron and other structural analogs. Likewise, CPR expression was not affected by these compounds as shown in the same figure. Given that the endogenous HO-1 expression in HEK293T cells is very low (Fig. 2d), additional experiment was performed to examine whether danthron and the structural analogs would downregulate the expression of HO-1. As demonstrated in the right panel of Fig. 3c, HO-1 level was not significantly reduced by the treatment with these compounds.

### Danthron inhibits physical interaction between HO-1 and CPR

**Fig. 4:**
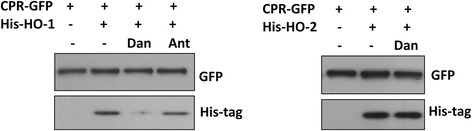
Danthron interferes the physical interaction of HO-1 but not HO-2 with CPR. Cell lysate was prepared from HEK293T cells transfected with CPR-GFP construct for 24 h. CPR-GFP protein was then immuno-precipitated by GFP-antibody-conjugated resin, followed by incubation with His-HO-1 or HO-2 protein in the absence or presence of 10 μM indicated compounds at room temperature for 1 h with rotation. The HO protein pulled down by CPR-GFP was then examined by Western blot analysis

To confirm the results obtained from BRET assay, we performed the pull down assay to assess the physical interaction between HO and CPR. CPR-GFP proteins overexpressed in HEK293T cells were first isolated by anti-GFP affinity resin, followed by incubation with recombinant soluble His-tagged HO-1 (His-HO-1) or His-tagged HO-2 (His-HO-2) protein in the absence or presence of 10 μM of danthron or anthrarufin as indicated for 1 h at room temperature. His-tagged HO-1 or HO-2 proteins interacting with CPR bound to anti-GFP affinity resin were then pulled down and examined by Western blot analysis. As demonstrated in Fig. 4, His-HO-1 and His-HO-2 proteins were pulled down by CPR-GFP immunoprecipitates, indicating the physical interaction of HO with CPR. Nevertheless, danthron co-incubation substantially reduced the binding of His-HO-1, but not His-HO-2 to CPR-GFP. Similar to that observed in BRET assay, anthrarufin treatment did not significantly affect the interaction between His-HO-1 and CPR-GFP as shown in the same figure.

### Danthron but not anthrarufin attenuates HO-1-mediated cancer cell growth and migration

**Fig. 5:**
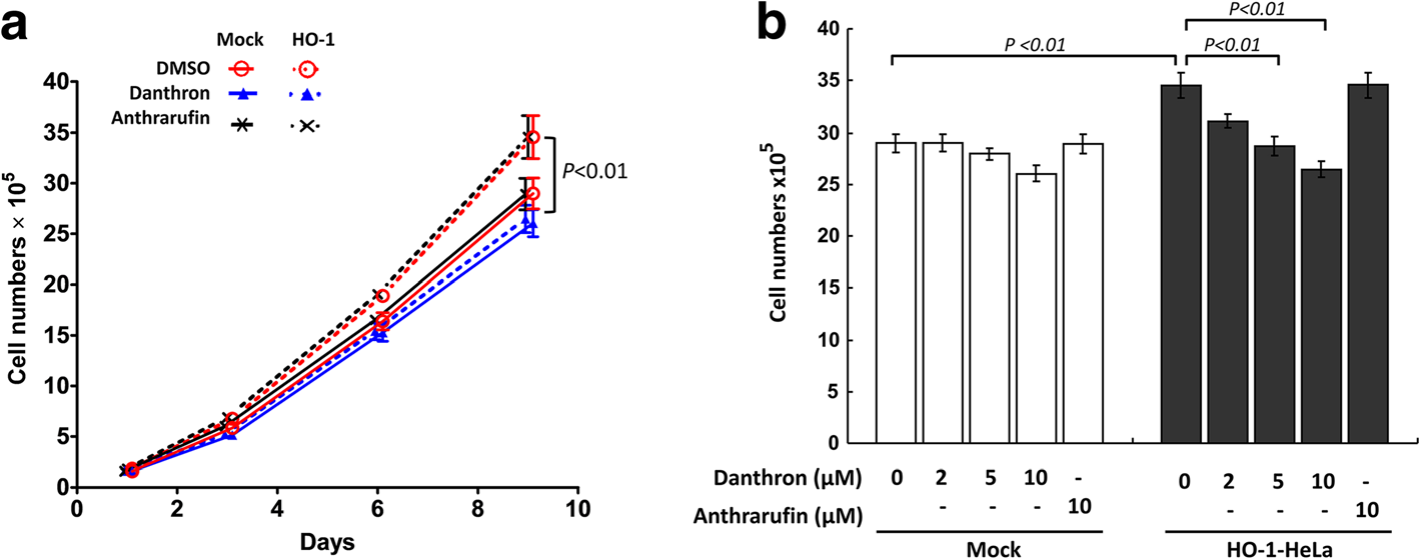
Danthron but not anthrarufin suppresses HO-1-induced cancer cell proliferation. **a** The growth rates of mock and HO-1-overexpressing HeLa cells cultured in the absence or presence of 10 μM indicated compounds were assessed. Data shown are the mean ± SE of three independent experiments. **b** The numbers of mock and HO-1-overexpressing cells cultured in the absence or presence of indicated concentrations of danthron or anthrarufin for 9 days were determined. Data shown are the mean ± SE of three independent experiments

**Fig. 6:**
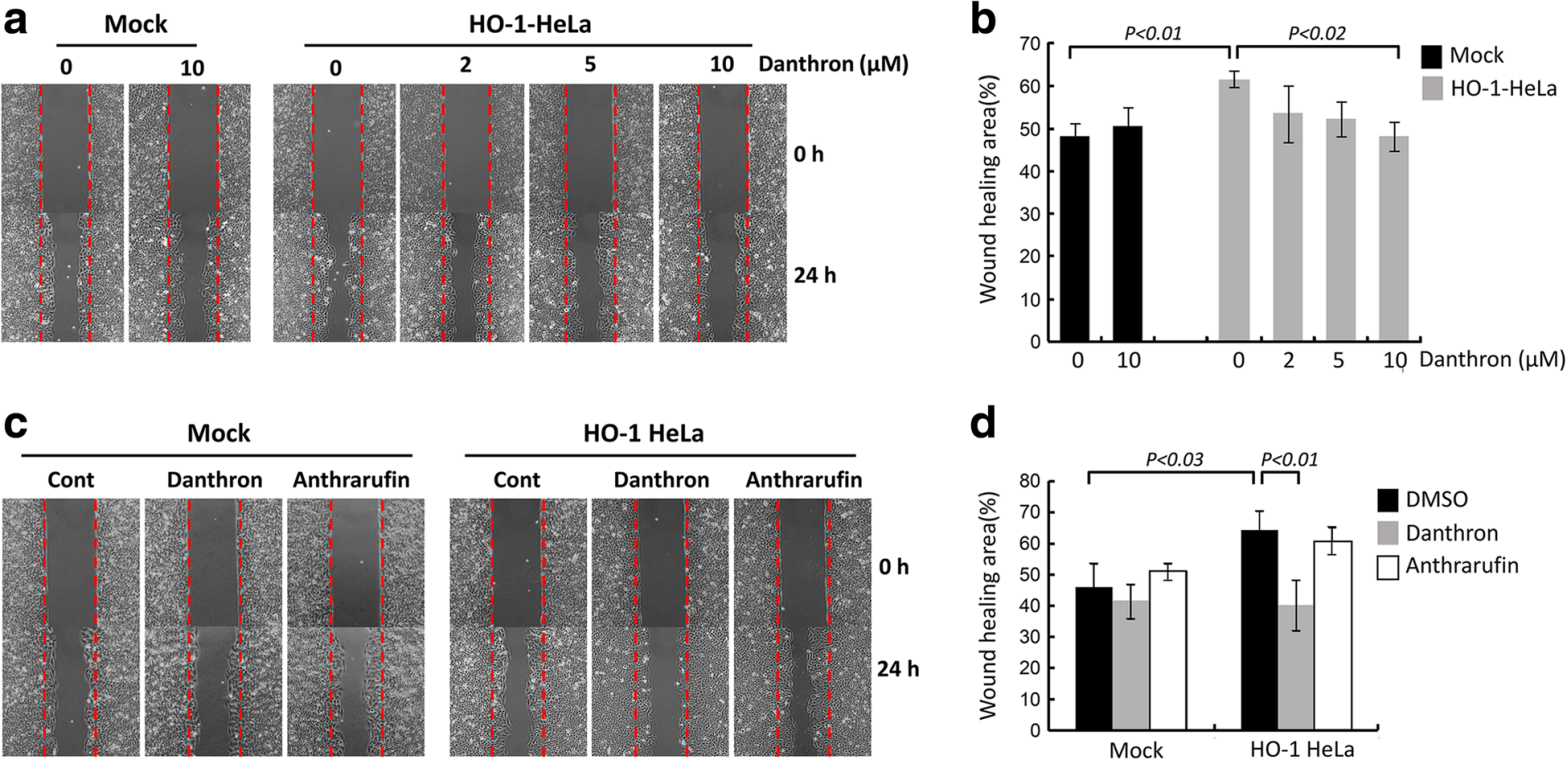
Danthron attenuates HO-1-induced cancer cell migration. **a** and **b** Wounds were made in confluent mock or HO-1 over-expressing HeLa cells and the cell migration toward wound area in the absence or presence of indicated concentration of danthron was examined at 24 h. Each treatment was performed in triplicate. The representative images are shown in a. The wound healing area of each treated group was quantified and shown in b. Data are the mean ± SE of three independent experiments. **c** and **d** Wound healing assays were performed in mock or HO-1 over-expressing HeLa cells in the absence or presence of 20 μM of indicated compounds as described above. The representative images are shown in c. The wound healing area of each treated group was quantified and shown in d. Data shown are the mean ± SE of three independent experiments

HO-1 overexpression has been shown to promote cancer cell growth and invasiveness [23]. We then performed experiments to examine whether disruption of the interaction between HO-1 and CPR has an impact on the HO-1-mediated effect on cancer cells. To this end, the effects of danthron and anthrarufin on the proliferation rates of mock and HO-1 overexpressing HeLa cell lines were assessed. As shown in Fig. 5a and b, the growth of mock cells was not significantly affected by the treatment with either compound at concentration of 10 μM, the highest concentration tested. However, the growth rate of HO-1-overexpressing Hela cells was significantly reduced by treatment with danthron dose-dependently. In contrast, anthrarufin at 10 μM had no significant effect on the growth of HO-1-overexpressing cells. When the cell migration was examined using a wound healing assay, the results showed that danthron treatment attenuated the migration response of HO-1-overexpressing cells but not mock cells in a dose-dependent manner (Fig. 6a and b). Again, anthrarufin did not have significant effect on both mock and HO-1-overexpressing cell lines even at concentration of 20 μM (Fig. 6c and d) These results demonstrate that interruption of HO-1/CPR interaction is able to attenuate the pro-tumorigenic effect of HO-1 in cancer cells.

## Discussion

Danthron is a natural anthraquinone derivative previously used as a laxative in humans. It was later shown to exhibit carcinogenic activity by inducing DNA damage and inhibiting DNA repair in rodents [26–28]. Nevertheless, danthron has been shown to function as a specific retinoic receptor antagonist [29] and regulates lipid and glucose metabolism by activating AMP-activated kinase [30]. Furthermore, there was a study showing that danthron induces apoptosis of glioma cells by increasing production of reactive oxygen species and decreasing mitochondrial membrane potential l [31]. Danthron and structural analogs also exhibit anti-oxidative and anti-inflammatory activities [32] and are novel inhibitors of scavenger receptor A expressed on macrophages [33]. These findings demonstrate that danthron and its structural analogs have multiple activities in various cellular contexts.

By conducting chemical library screening with a cell-based BRET assay, we identified danthron as a specific inhibitor to interfere the interaction of CPR with HO-1, but not with HO-2. The isoform-specific effect of danthron on CPR-HO-1 interaction was also confirmed by pull-down assay. HO-1 and HO-2 are HO isoforms derived from two distinct gene products. Sequence alignment shows that HO-1 and HO-2 share about 50% homology in primary amino acid sequences [1]. However, they share high structural similarity, especially the conserved heme binding region [9]. The chance for same inhibitor targeting to the catalytic sites of both HO isozymes is high. Therefore, the development of isoform-specific inhibitor for HO-1, the inducible form highly expressed in various pathological states, is a difficult task. The present finding provide the potential to design inhibitors to block the function of HO-1 without influencing HO-2. Further BRET assays revealed that danthron reduced BRETmax but not BRET_50_ of CPR-HO-1 interaction, indicating that it affects the distance or relative orientations between CPR and HO-1 but not affecting their binding affinity. Although dantron could reduce over 80% of the physical interaction between HO-1 and CPR in pull-down assay, the degree of inhibition assessed by cell-based BRET assay appeared to be much less. It is likely due to the fact that the drug treatment in BRET assay was performed with preexisting HO-1/CPR complex in cells. Furthermore, the possibility that the activity of danthron is modulated by other endogenous factors in cellular context cannot be completely ruled out. Although the molecular structure of CPR/HO-1/danthron complex was not determined in present study, we showed that the structural analogs of danthron bearing hydroxyl groups at different positions of the phenolic rings affected CPR/HO-1 interaction with various potencies. Anthrarufin, which has two hydroxyl groups located in 1,5 positions of anthraquinone, failed to interfere CPR/HO-1 interaction as demonstrated by BRET assay. Again, none of the structural analogs tested affects CPR/HO-2 interaction. These observations highlight the importance of structural feature in danthron-mediated inhibition of CPR/HO-1 interaction.

To further explore whether the disruption of CPR/HO-1 interaction results in the ablation of HO-1-mediated cellular effects, the effects of danthron and anthrarufin on HO-1-enhanced proliferation and migration responses of HeLa cells were examined. The results clearly showed that danthron, but not anthrarufin, does-dependently suppressed the increases in growth rate and migration in HO-1-overexpressing cells without a significant effect on mock control cells. Given that HO-1 is overexpressed in many cancers and implicated in tumor progression and resistance to chemotoxic agents and irradiation [4–8], the present finding supports the therapeutic potential of developing inhibitors of CPR-HO-1 interaction for cancer treatment.

## Conclusions

It is apparent that danthron exhibits paradoxical activities on cancer. Earlier studies have revealed the carcinogenic activity of danthron in normal cells, resulting in the suspension of its clinical use. Nevertheless, the present study demonstrate that danthron has anti-tumorigenic effect via interfering the interaction of CPR with HO-1 which is highly induced in many types of cancer with pro-tumorigenic function. Notably, danthron does not affect the interaction between CPR and HO-2. Although danthron cannot be considered as a candidate drug for cancer therapy, the present study supports the possibility of using danthron as a lead compound to design better isoform-specific inhibitors to block HO-1 activity with higher potency.

## Abbreviations

BRET: Bioluminescence resonance energy transfer
CO: Carbon monoxide
CPR: Cytochrome P450 reductase
GFP: Green fluorescent protein
HO: Heme oxygenase
Luc: Luciferase

## References

[1] Maines MD (1988). Heme oxygenase: function, multiplicity, regulatory mechanisms, and clinical applications. FASEB J.

[2] Gutierrez A, Grunau A, Paine M, Munro AW, Wolf CR, Roberts GC (2003). Electron transfer in human cytochrome P450 reductase. Biochem Soc Trans.

[3] Abraham NG, Kappas A (2008). Pharmacological and clinical aspects of heme oxygenase. Pharmacol Rev.

[4] Chau LY (2015). Heme oxygenase-1: emerging target of cancer therapy. J Biomed Sci.

[5] Miyake M, Fujimoto K, Anai S, Ohnishi S, Nakai Y (2010). Inhibition of heme oxygenase-1 enhances the cytotoxic effect of gemcitabine in urothelial cancer cells. Anticancer Res.

[6] Liu YS, Li HS, Qi DF, Zhang J, Jiang XC (2014). Zinc protoporphyrin IX enhances chemotherapeutic response of hepatoma cells to cisplatin. World J Gastroenterol.

[7] Berberat PO, Dambrauskas Z, Gulbinas A, Giese T, Giese N (2005). Inhibition of heme oxygenase-1 increases responsiveness of pancreatic cancer cells to anticancer treatment. Clin Cancer Res.

[8] Kongpetch S, Kukongviriyapan V, Prawan A, Senggunprai L, Kukongviriyapan U (2012). Crucial role of heme oxygenase-1 on the sensitivity of cholangiocarcinoma cells to chemotherapeutic agents. PLoS One.

[9] Rahman MN, Vukomanovic D, Vlahakis JZ, Szarek WA, Nakatsu K (2013). structural insights into human heme oxygenase-1 inhibition by potent and selective azole-based compounds. J R Soc Interface.

[10] Salerno L, Pittala V, Romeo G, Modica MN, Siracusa MA (2013). Evaluation of novel aryloxyalkyl derivatives of imidazole and 1,2,4-triazole as heme oxygenase-1 (HO-1) inhibitors and their antitumor properties. Bioorg Med Chem.

[11] Pittala V, Salerno L, Romeo G, Modica MN, Siracusa MA (2013). A focus on heme oxygenase-1 (HO-1) inhibitors. Curr Med Chem.

[12] Nero TL, Morton CJ, Holien JK, Wielens J, Parker MW (2014). Oncogenic protein interfaces: small molecules, big challenges. Nat Rev Cancer.

[13] Jin L, Wang W, Fang G (2014). Targeting protein-protein interaction by small molecules. Annu Rev Pharmacol Toxicol.

[14] Ferreira LG, Oliva G, Andricopulo AD (2016). Protein-protein interaction inhibitors: advances in anticancer drug design. Expert Opin Drug Discov.

[15] Wang J, de Montellano PR (2003). The binding sites on human heme oxygenase-1 for cytochrome p450 reductase and biliverdin reductase. J Biol Chem.

[16] Higashimoto Y, Sakamoto H, Hayashi S, Sugishima M, Fukuyama K (2005). Involvement of NADPH in the interaction between heme oxygenase-1 and cytochrome P450 reductase. J Biol Chem.

[17] Higashimoto Y, Sugishima M, Sato H, Sakamoto H, Fukuyama K (2008). Mass spectrometric identification of lysine residues of heme oxygenase-1 that are involved in its interaction with NADPH-cytochrome P450 reductase. Biochem Biophys Res Commun.

[18] Bacart J, Corbel C, Jockers R, Bach S, Couturier C (2008). The BRET technology and its application to screening assays. Biotechnol J.

[19] Couturier C, Deprez B (2012). Setting up a bioluminescence resonance energy transfer high throughput screening assay to search for protein/protein interaction inhibitors in mammalian cells. Front Endocrinol.

[20] Hwang HW, Lee JR, Chou KY, Suen CS, Hwang MJ, Chen C (2009). Oligomerization is crucial for the stability and function of heme oxygenase-1 in the endoplasmic reticulum. J Biol Chem.

[21] Cheng YH, Ho MS, Huang WT, Chou YT, King K (2015). Modulation of glucagon-like Peptide-1 (GLP-1) potency by endocannabinoid-like lipids represents a novel mode of regulating GLP-1 receptor signaling. J Biol Chem.

[22] Lin PH, Chiang MT, Chau LY (2008). Ubiquitin-proteasome system mediates heme oxygenase-1 degradation through endoplasmic reticulum-associated degradation pathway. Biochim Biophys Acta.

[23] Hsu FF, Yeh CT, Sun YJ, Chiang MT, Lan WM (2015). Signal peptide peptidase-mediated nuclear localization of heme oxygenase-1 promotes cancer cell proliferation and invasion independent of its enzymatic activity. Oncogene.

[24] Borroto-Escuela DO, Flajolet M, Agnati LF, Greengard P, Fuxe K (2013). Bioluminescence resonance energy transfer methods to study G protein-coupled receptor-receptor tyrosine kinase heteroreceptor complexes. Methods Cell Biol.

[25] Guan R, Feng X, Wu X, Zhang M, Zhang X (2009). Bioluminescence resonance energy transfer studies reveal constitutive dimerization of the human lutropin receptor and a lack of correlation between receptor activation and the propensity for dimerization. J Biol Chem.

[26] National Toxicology P (2002). Danthron (1,8-dihydroxyanthraquinone). Rep Carcinog.

[27] Sjoberg P, Hedelin U, Kronevi T, Lyden-Sokolowski A, Magnusson G (1988). Pigmentation of kidneys and lymph nodes of mesocolon in rats fed diets containing the laxative danthron. Toxicol Lett.

[28] Mueller SO, Stopper H (1999). Characterization of the genotoxicity of anthraquinones in mammalian cells. Biochim Biophys Acta.

[29] Zhang H, Zhou R, Li L, Chen J, Chen L (2011). Danthron functions as a retinoic X receptor antagonist by stabilizing tetramers of the receptor. J Biol Chem.

[30] Zhou R, Wang L, Xu X, Chen J, Hu LH (2013). Danthron activates AMP-activated protein kinase and regulates lipid and glucose metabolism in vitro. Acta Pharmacol Sin.

[31] Chiou SM, Chiu CH, Yang ST, Yang JS, Huang HY (2012). Danthron triggers ROS and mitochondria-mediated apoptotic death in C6 rat glioma cells through caspase cascades, apoptosis-inducing factor and endonuclease G multiple signaling. Neurochem Res.

[32] Nam W, Kim SP, Nam SH, Friedman M. Structure-Antioxidative and anti-inflammatory activity relationships of Purpurin and related Anthraquinones in chemical and cell assays. Molecules. 2017; 10.3390/molecules22020265. 10.3390/molecules22020265 PMC6155578 28208613

[33] Zheng Y, Li X, Pagare PP, Yuan Y, Wang XY, Zhang Y (2017). Design, synthesis, and characterization of rhein analogs as novel inhibitors of scavenger receptor a. Bioorg Med Chem Lett.

